# The Diphtheria Toxin Translocation Domain Impairs Receptor Selectivity in Cancer Cell-Targeted Protein Nanoparticles

**DOI:** 10.3390/pharmaceutics14122644

**Published:** 2022-11-29

**Authors:** Eric Voltà-Durán, Julieta M. Sánchez, Eloi Parladé, Naroa Serna, Esther Vazquez, Ugutz Unzueta, Antonio Villaverde

**Affiliations:** 1 Institut de Biotecnologia i de Biomedicina, Universitat Autònoma de Barcelona, Bellaterra, 08193 Barcelona, Spain; 2 CIBER de Bioingeniería, Biomateriales y Nanomedicina (CIBER-BBN), Instituto de Salud Carlos III, Bellaterra, 08193 Barcelona, Spain; 3 Departament de Genètica i de Microbiologia, Universitat Autònoma de Barcelona, Bellaterra, 08193 Barcelona, Spain; 4 Instituto de Investigaciones Biológicas y Tecnológicas (IIBYT), CONICET-Universidad Nacional de Córdoba, Av. Velez Sarsfield 1611, Córdoba X5016GCA, Argentina; 5 Institut d’Investigació Biomèdica Sant Pau (IIB SANT PAU), Sant Quintí 77-79, 08041 Barcelona, Spain; 6 Josep Carreras Leukaemia Research Institute, 08025 Barcelona, Spain

**Keywords:** recombinant proteins, nanoparticles, diphtheria toxin, self-assembling, cell-penetrating peptide

## Abstract

Protein-based materials intended as nanostructured drugs or drug carriers are progressively gaining interest in nanomedicine, since their structure, assembly and cellular interactivity can be tailored by recruiting functional domains. The main bottleneck in the development of deliverable protein materials is the lysosomal degradation that follows endosome maturation. This is especially disappointing in the case of receptor-targeted protein constructs, which, while being highly promising and in demand in precision medicines, enter cells via endosomal/lysosomal routes. In the search for suitable protein agents that might promote endosome escape, we have explored the translocation domain (TD) of the diphtheria toxin as a functional domain in CXCR4-targeted oligomeric nanoparticles designed for cancer therapies. The pharmacological interest of such protein materials could be largely enhanced by improving their proteolytic stability. The incorporation of TD into the building blocks enhances the amount of the material detected inside of exposed CXCR4^+^ cells up to around 25-fold, in absence of cytotoxicity. This rise cannot be accounted for by endosomal escape, since the lysosomal degradation of the new construct decreases only moderately. On the other hand, a significant loss in the specificity of the CXCR4-dependent cellular penetration indicates the unexpected role of the toxin segment as a cell-penetrating peptide in a dose-dependent and receptor-independent fashion. These data reveal that the diphtheria toxin TD displayed on receptor-targeted oligomeric nanoparticles partially abolishes the exquisite receptor specificity of the parental material and it induces nonspecific internalization in mammalian cells.

## 1. Introduction

Protein drugs benefit from the structural versatility and broad and multiple functionality of natural polypeptides, whose activities cover most biological events in living systems [1,2,3]. In addition, protein functions can be tuned by genetic engineering and combined in single-chain modular proteins [4]. Therefore, apart from plain protein drugs, such as insulin versions, interferons, hormones, antibodies and enzymes, the possibility to combine, for instance, a cell-targeting domain and a cytotoxic protein in a single molecular product is the basis for ongoing research in many clinical areas, especially in oncology [5]. In this context, fusion proteins can be designed to recruit complementing capabilities in multifunctional constructs [4,6], whose active segments are selected from the vast catalogue of functional protein domains so far identified. Since proteins are produced at an industrial scale as recombinant versions in a diversity of cell factories, protein drugs are progressively increasing their presence in the list of approved pharmaceuticals [2,7,8,9].

**Figure 1:**
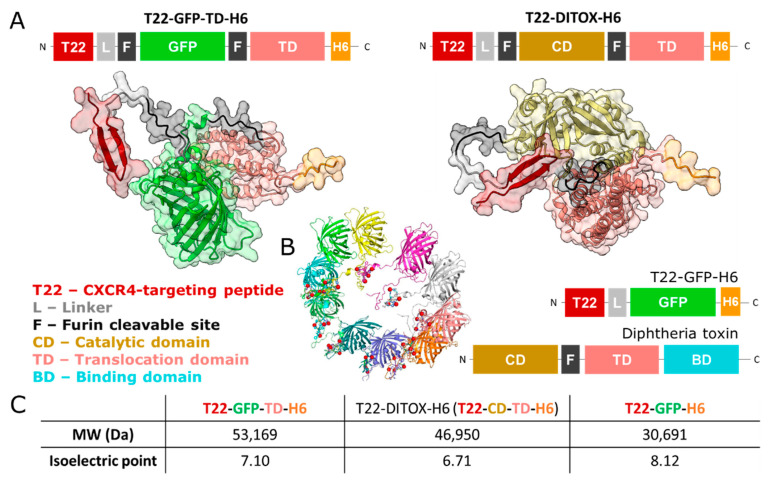
Modular organization of T22-empowered proteins. (**A**) Modular disposition and 3D models of T22-GFP-TD-H6 and T22-DITOX-H6. T22-GFP-TD-H6 was engineered to mimic the CXCR4-targeted toxin T22-DITOX-H6 through the replacement of the catalytic domain of the toxin (CD) by the green fluorescent protein (GFP). The translocation domain (TD) was maintained. Both proteins display, from the N to the C terminus, the CXCR4 ligand T22, a flexible linker (L; GGSSRSS), two furin cleavable sites (F; GNRVRRSV) recruited from the original DT, and the hexa-histidine tag (H6), all represented in color code. Furin cleavable sites have proved to be functional in this family of modular constructions [48]. Models in 3D were predicted in AlphaFold2 and represented with ChimeraX. (**B**) The modular disposition of the DT and T22-GFP-H6 are also shown as references. In the center is the T22-GFP-H6 nanoparticle as previously modelled [49], with each monomer represented in a different color and the stabilizing divalent cations as red spheres (reprinted from [49] with permission from Elsevier). (**C**) Molecular weight and isoelectric point of the three proteins used in this study, predicted by ProtParam (Expasy, http://web.expasy.org/protparam; accessed on 20 November 2022).

Among the main obstacles for the use of drug proteins are their potential immunogenicity (when from non-human sources) and the proteolytic degradation in the lysosomal compartment. De-immunization by site-directed mutagenesis and other engineering strategies have allowed for a large reduction in immunotoxicities, such as the ones reported in several interesting products [10,11,12,13,14]. However, lysosomal degradation remains the main bottleneck in the design of effective protein drugs intended for intracellular activities [15,16,17,18]. Regarding the emerging interest in receptor-mediated, cell-targeted drug delivery in oncology and other fields (aimed to enhance specificity and to reduce non-desired side-effects) [19,20,21], endosomal uptake of protein drugs results in the degradation of most of the internalized product and in the consequent reduction of effectivity [15,18,22,23]. In this context, endosomal-escape protein segments are desirable components of protein-based drugs. Among these peptides, short amino acid sequences (for instance, histidine-rich peptides) or more complex protein domains, some of them from pathogenic microorganisms, have been explored and/or adapted with different levels of success to protein constructs aimed at cell-targeted delivery [16,18,22,24,25]. In particular, the diphtheria toxin (DT) produced by *Corynebacterium diphtheriae* kills mammalian cells by inactivating the elongation factor EF-2 [26]. This protein exhibits a modular architecture (Figure 1A) in which the translocation domain (TD, also called T domain) plays a critical role in allowing the catalytic domain (CD) to reach the cytosol from endosomal compartments [27,28]. At a late endosome acidic pH, the DT TD undergoes conformational changes that drive its insertion in the endosomal membrane, resulting in the formation of a small pore by which the CD is transported to the cytosol. The DT TD has been used for the active translocation of protein modules to the cytosol, proving its ability to assist in the membrane crossing of polypeptides different from CD [29,30]. Although the precise mechanism by which proteins are translocated to the cytosol by the DT TD is a controversial subject [31,32], the DT TD, free from the active catalytic domain, it is an agent of potential interest in drug delivery [33,34]. In addition, as far as we know, there are no existing reports about the potential role of TD in promoting cell surface internalization previous to receptor-mediated endosomal uptake. We have explored here the applicability of the DT TD as a functional component in self-assembling protein-only nanoparticles targeted to the cell surface cytokine receptor CXCR4, which is a relevant molecular marker for anti-cancer drug targeting [35,36,37,38]. The highly precise and selective targeting is provided by the peptide T22, an exclusive ligand of CXCR4 [39,40,41]. This has been done through the incorporation of the TD into the well-characterized protein T22-GFP-H6, which self-assembles as regular homo-oligomeric nanoparticles of 12 nm and is used as a nanoscale drug vehicle in cancer therapies [42,43]. T22-GFP-H6 enters cells via the surface-exposed cytokine receptor CXCR4, but it undergoes important lysosomal degradation estimated to affect between 85–90% of the engulfed material [25]. While the extent of protein destruction may only moderately affect the activities of attached conventional chemical drugs, such as floxuridine, cytarabine or auristatin E [43,44,45,46], it might be a limitation for the therapeutic outcome of protein-only pharmaceuticals [5], including H6-tagged versions with self-assembling properties [47].

## 2. Materials and Methods

### 2.1. Genetic Design and Protein Production and Purification

**Figure 2:**
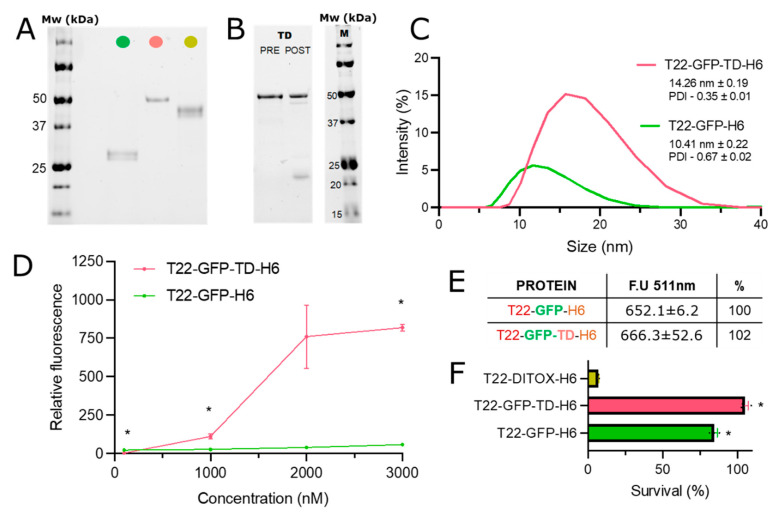
Characterization of T22-GFP-TD-H6 nanoparticles and their cellular accumulation. (**A**) Protein gel electrophoresis of the three proteins used in the study (T22-GFP-H6, in green; T22-GFP-TD-H6, in dark pink; T22-DITOX-H6, in dark yellow). (**B**) Protein gel electrophoresis of T22-GFP-TD-H6, before (pre) and after (post) exposure to recombinant furin. (**C**) Intensity size distribution of T22-GFP-H6 and T22-GFP-TD-H6, measured by Dynamic Light Scattering (DLS). Inset values indicate mean size measured by intensity (nm) and polydispersity index (PDI) ± standard error. (**D**) Relative intracellular accumulation of T22-GFP-H6 and T22-GFP-TD-H6 in HeLa cells after 24 h of exposure, at four different concentrations (100 nM, 1000 nM, 2000 nM, 3000 nM). Data are shown as mean ± standard error. Fluorescence values are referred to those of background cells. Significant statistical differences between data pairs (T22-GFP-H6 vs. T22-GFP-TD-H6 at a respective concentration) are represented by * at *p* < 0.05. (**E**) Intrinsic fluorescent emission of T22-GFP-H6 and T22-GFP-TD-H6, exciting at 488 nm and detecting at 511 nm. Fluorescence of T22-GFP-H6 is stated as 100%. (**F**) HeLa cell viability upon exposure to 1000 nM of T22-GFP-H6, T22-GFP-TD-H6 and T22-DITOX-H6 for 48 h. Data are shown as mean ± standard error. Statistical significance with respect to the toxin group (T22-DITOX-H6) is represented by * *p* < 0.05.

*Escherichia coli* Origami B (BL21, OmpT−, Lon−, TrxB−, Gor−) (Novagen) were used to produce T22-GFP-H6, T22-GFP-TD-H6 and T22-DITOX-H6 (Figure 1A). T22-GFP-TD-H6 gene was designed in-house as codon-optimized genes for *E. coli*, synthetized and inserted in pET22b plasmids by Geneart (Thermo Fisher Scientific, Waltham, MA, USA). T22-GFP-TD-H6 was conceived to include, from the N-terminus to the C-terminus, the following modules: T22, a peptide that targets the cytokine receptor CXCR4 [38]; a flexible linker (GGSSRSS), to provide structural flexibility to T22; the DT furin cleavable site (F, GNRVRRSV); the green fluorescent protein (GFP); a second furin cleavable site (F); the DT translocation domain (TD); and the hexa-histidine (H6) tag. The H6 tag was used for both single-step Ni^2+^-based affinity chromatography purification and for ion-assisted assembly of the monomers into oligomeric nanoparticles, because of the cross-linking properties of H6 [47]. Origami B cells were transformed by heat shock, grown in LB medium (supplemented with 1 × 10^−1^ g·L^−1^ ampicillin, 1.25 × 10^−2^ g·L^−1^ tetracycline and 1.5 × 10^−2^ g·L^−1^ kanamycin). T22-GFP-TD-H6 was produced at 20 °C overnight upon induction with 1 × 10^−3^ M isopropyl β-D-1-thiogalactopyranoside (IPTG). Two rounds of French Press (Thermo Fisher Scientific FA-078A, Waltham, MA, USA) at 18,000 psi were applied to obtain bacterial lysates, in the presence of a wash buffer (20 mmol L^−1^ Tris-HCl, 500 mmol L^−1^ NaCl, 10 mmol L^−1^ imidazole, pH 8.0) and a protease inhibitor cocktail (cOmplete EDTA-free, Roche Diagnostics, Basel, Switzerland). Soluble fractions were collected by centrifugation (45 min, 15,000× *g*). T22-GFP-TD-H6 was purified by immobilized metal affinity chromatography (IMAC) using HisTrap HP 1 mL columns (Cytiva, Marlborough, MA, USA). Elution was achieved by a linear gradient of elution buffer (2 × 10^−2^ M Tris-HCl, 5 × 10^−1^ M NaCl, 1 M imidazole, pH 8.0) and the purified protein was finally dialyzed against sodium bicarbonate (166 × 10^−3^ M NaHCO_3_, pH 8.0) with or without sodium chloride (333 × 10^−3^ M NaCl). T22-GFP-H6 and T22-DITOX-H6 were produced and purified by slight variations of this protocol. Precise details can be found elsewhere [48]. The oligomeric organization of T22-GFP-H6 is shown in Figure 2B and the main proteomic traits of all these constructs are depicted in Figure 1C. 

### 2.2. Three-Dimensional Protein Modelling and Visualization

An in silico three-dimensional structure prediction of T22-GFP-TD-H6 and T22-DITOX-H6 was performed using AlphaFold2 [50] via ColabFold [51]. ChimeraX software (version 1.2) was used for their 3D structure visualization [52]. The surface was shown with a transparency of 0.8.

### 2.3. Protein Purity, Integrity and Concentration

Protein purity and integrity was assessed by sodium dodecyl sulphate polyacrylamide gel electrophoresis (SDS-PAGE), anti-His (Santa Cruz Biotechnology, Dallas, TX, USA) western blot immunodetection (not shown) and matrix-assisted laser-desorption ionization time-of-flight (MALDI-TOF, not shown). Protein concentration was determined by Bradford assay.

### 2.4. Size Distribution and Fluorescence Emission

The size distribution of T22-GFP-H6 and T22-GFP-TD-H6 was determined by dynamic light scattering (DLS) in a Zetasizer Nano ZS (Malvern Panalytical, Malvern, UK) at 633 nm and 25 °C, using a ZEN2112 3 mm quartz cuvette. Measurements were performed in triplicate. Mean hydrodynamic size and standard error of the mean (SEM) were obtained from these values. The polydispersity index was provided directly by the equipment. The green fluorescence emission of T22-GFP-H6 and T22-GFP-TD-H6 was measured in a Varian Cary eclipse fluorescence spectrophotometer (Agilent Technologies, Santa Clara, CA, USA), exciting at 488 nm (emission peak at 511 nm). Measurements were performed in duplicate. All protein solutions were used at the same concentration (1 g·L^−1^). Values were then corrected according to their molecular weight.

### 2.5. Cell Culture and Confocal Laser Scanning Microscopy

The performance of T22-GFP-TD-H6 was studied in vitro in CXCR4^+^ HeLa (ATCC-CCL2) cells and CXCR4^−^ SW1417 (ATCC-CCL238) cells. The differential surface CXCR4 levels in these cell lines have been previously confirmed [53]. HeLa cells (ATCC-CCL2) were maintained in Eagle minimum essential medium (MEM Alpha 1× GlutaMAX^TM^, Gibco^TM^ Thermo Fisher Scientific, Waltham, MA, USA) supplemented with 10% foetal bovine serum at 37 °C in a 5% CO_2_ humidified atmosphere. For confocal microscopy, HeLa cells were grown on MatTek plates (MatTek Corporation, Ashland, MA, USA) at 50,000 cells/plate, during 24 h. Then, MEM Alpha was replaced by OptiPro^TM^ serum-free medium (Gibco^TM^ Thermo Fisher Scientific, Waltham, MA, USA) and cells were exposed to T22-GFP-TD-H6 or T22-GFP-H6 at 1000 nM for 2 additional hours. Before imaging, plates were washed with phosphate buffer saline (PBS) and subsequently labelled with Hoechst 33342 (Thermo Fisher Scientific, Waltham, MA, USA) and CellMask^TM^ Deep Red (Thermo Fisher Scientific, Waltham, MA, USA), at 5 × 10^−3^ g·L^−1^ and 2.5 × 10^−3^ g·L^−1^, respectively. Cells were then washed with PBS and visualized in Mem Alpha, using an inverted ZEISS LSM 980 inverted Confocal Laser Scanning Microscope with Airyscan 2 detector (Carl Zeiss Microscopy GmbH, Jena, Germany) with 63× (1.4 NA) oil immersion objective lenses. For Hoechst 33342, excitation was reached via a 405 nm diode laser; for GFP, via a 488 nm diode laser; and for CellMask^TM^ Deep Red, via a 639 nm diode laser. The confocal pinhole was set to 4.72 AU for GFP, 7.12 AU for Hoechst 33342 and 5.50 AU for CellMask^TM^ Deep Red. The detector gain was set to 670 V. Multitrack sequential acquisition settings were used. Emission detection bandwidths were optimized and configured to avoid cross-talking. Images were processed using ZEN 3.5 (ZEN Lite) software (Carl Zeiss Microscopy GmbH, Jena, Germany). For 3D reconstructions, z-stacks were acquired every 0.5 µm. Images were then processed using the Surpass Module from Imaris v 7.2.1 software (Bitplane, Belfast, UK).

### 2.6. Cell Cytometry

To monitor the internalization of nanoparticles, HeLa cells exposed to different protein concentrations (from 1 × 10^−7^ M to 3 × 10^−6^ M) for different times (from 30 min to 24 h) were analyzed by flow cytometry using a fluorescence-assisted cell sorting (FACS)-Canto system (Becton Dickinson, Franklin Lakes, NJ, USA) using a 15 mW air-cooled argon ion laser exciting at 488 nm. All measurements were performed in duplicate. HeLa cells were seeded in 24-well culture plates at 30,000 cells/well. Proteins were incubated in presence of OptiPro SFM (Gibco^TM^ Thermo Fisher Scientific, Waltham, MA, USA) supplemented with L-glutamine, at 37 °C in a 5% CO_2_ humidified atmosphere. After protein exposure, Trypsin-EDTA 1 mg/mL (Gibco^TM^ Thermo Fisher Scientific, Waltham, MA, USA) was applied to detach cells with a harsh protocol specifically designed to remove externally attached proteins ([54], 15 min, 37 °C). The displayed relative fluorescence values were obtained by dividing the intracellular fluorescence values at each condition by the background fluorescence values of non-exposed control cells in the same experiment. To determine receptor-specificity in protein internalization, the intracellular fluorescence of HeLa cells exposed to T22-GFP-H6 or T22-GFP-TD-H6 (at 1 × 10^−6^ M and 2 × 10^−6^ M for 1 h) was compared to the intracellular fluorescence of HeLa cells exposed to the same protein in the same conditions, previously incubated with the CXCR4 antagonist AMD3100 octahydrochloride hydrate (Sigma-Aldrich, San Luis, MO, USA) [55,56,57] for 1 h, at a final concentration ten-fold the protein concentration (1 × 10^−5^ M or 2 × 10^−5^ M). Specificity (as %) was calculated as 100 × (1 − A/B), in which A refers to intracellular fluorescence values in presence of AMD3100 (non-specific internalization) and B refers to intracellular fluorescence values in absence of AMD3100. All measurements were performed in duplicate.

CXCR4^−^ SW1417 cells were used to determine the intracellular CXCR4-independent accumulation of T22-GFP-TD-H6. SW1417 cells were grown in Dulbecco’s Eagle minimum essential medium (DMEM Alpha 1x GlutaMAX^TM^, Gibco^TM^ Thermo Fisher Scientific, Waltham, MA, USA). SW1417 cells were seeded in 24-well culture plates at 180,000 cells/well and were exposed for 24 h at different concentrations of T22-GFP-TD-H6 (from 1 × 10^−7^ M to 2 × 10^−6^ M) in presence of OptiPro SFM supplemented with L-glutamine, at 37 °C in a 10% CO_2_ humidified atmosphere. Samples were analysed by flow cytometry using a Cytoflex (Beckman Counter, Brea, CA, USA) with a 50 mW solid state laser exciting at 488 nm, following the harsh trypsinization protocol mentioned above. All measurements were performed in duplicate.

To evaluate endosomal degradation of engulfed nanoparticles, the intracellular fluorescence of HeLa cells exposed to T22-GFP-H6 or T22-GFP-TD-H6 at 2 × 10^−6^ M for different times was compared to the intracellular fluorescence of HeLa cells exposed to the same protein at the same conditions, but previously incubated with the lysosomal degradation inhibitor chloroquine [58,59] (chloroquine diphosphate salt, Sigma-Aldrich, San Luis, MO, USA) (at a final concentration of 5 × 10^−4^ M). All measurements were performed in duplicate.

### 2.7. Cell Viability

To monitor protein cytotoxicity, HeLa cells were seeded (3.5 × 10^3^ cells/well) in Mem Alpha medium in 96-well opaque plates and grown for 24 h at 37 °C in a 5% CO_2_ humidified atmosphere. Cells were then exposed to T22-GFP-H6, T22-GFP-TD-H6 and T22-DITOX-H6 (positive control) for 48 h, at a final concentration of 2 × 10^−6^ M and a final volume of 1 × 10^4^ L. Finally, the Cell-Titer Glo Luminescent Cell Viability Assay (Promega Corporation, Madison, WI, USA) protocol was followed. A Victor3 (PerkinElmer, Waltham, MA, USA) microplate reader was used for the measurements. Experiments were performed in triplicate.

### 2.8. Proteolytic Analysis

The accessibility of the furin cleavable site was assessed by SDS-PAGE, after 6 h of reaction with a recombinant furin enzyme (New England Biolabs, Ipswich, MA, USA). The reaction took place at 25 °C in the reaction buffer recommended by the supplier (2 × 10^−2^ M HEPES, 0.1% Triton X-100, 1 × 10^−3^ M CaCl2, 2 × 10^−4^ M β-mercaptoethanol, pH 7.5). T22-GFP-TD-H6 was added at a final concentration of 10^−1^ g·L^−1^., in a final volume of 2.5 × 10^−5^ L with 0.1 units of enzyme.

### 2.9. Statistical Analysis

Mean comparisons were performed using the unpaired *t*-test. Statistical significance is represented by * (*p* value < 0.05). All statistical analyses were performed in GraphPad Prism 9.4.1 (GraphPad Software).

## 3. Results

It has been proposed that the formation of membrane pores mediated by the DT TD at acidic pH might allow the endosomal escape of a fraction of the engulfed polypeptides [29,60], but this functional domain had not been deeply characterized in finely cell targeted protein oligomers for precision medicines. In light of the need to combine precise cell targeting and endosomal escape, we constructed the modular protein T22-GFP-TD-H6 (Figure 1A) to test the cytosolic release of a reporter GFP in the CXCR4-targeted drug delivery vehicle T22-GFP-H6. The parental polypeptide T22-GFP-H6 spontaneously assembles, assisted by ions from the media, as regular 12 nm nanoparticles (a validated structural model of the T22-GFP-H6 nanoparticle is also shown in Figure 1B, [49]). This material mediates the selective delivery of covalently attached chemical drugs [45,46,61] and of proapoptotic peptides, placed as GFP fusions [62,63], into CXCR4^+^ cancer cells, in vitro and in vivo.

As in the case of T22-GFP-H6 and T22-DITOX-H6, T22-GFP-TD-H6 was well produced in *Escherichia coli* as a full-length soluble protein (Figure 2A), in which the furin cleavable sites were functional (Figure 2B). Upon purification, the protein was observed to assemble into monodisperse nanoparticles of around 14 nm, slightly larger than the parental T22-GFP-H6 (Figure 2C). Being fully fluorescent (Figure 2D), T22-GFP-TD-H6 oligomers could be easily monitored during their interaction with target cells. In this context, when CXCR4^+^ HeLa cells were exposed to T22-GFP-TD-H6, high levels of intracellular fluorescence were observed. Such protein amounts represented a dramatic increase (almost up to 20-fold) over those observed upon the exposure to the parental T22-GFP-H6 material (Figure 2D). Since the specific fluorescence of both proteins is comparable (Figure 2E), the fluorescent emission can be interpreted as a validated parameter to comparatively estimate the intracellular GFP amount. As in the case of T22-GFP-H6, such uptake occurred in absence of cytotoxicity (Figure 2F). This is in contrast to what occurred when exposing cells to the control nanoparticle T22-DITOX-H6, which accommodates the catalytic domain of the toxin instead GFP (Figure 2F).

**Figure 3:**
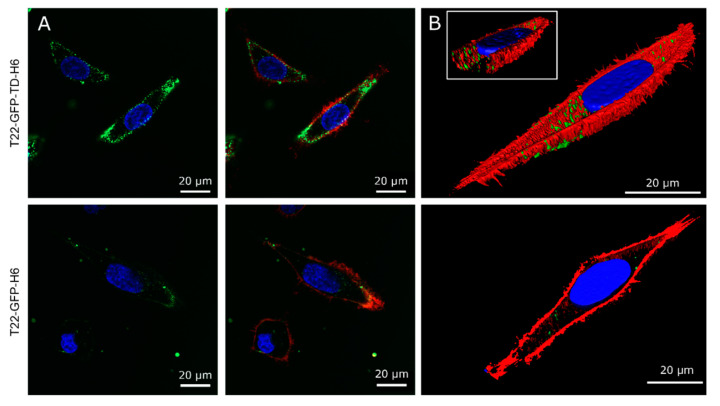
Intracellular distribution of T22-GFP-TD-H6 and T22-GFP-H6. (**A**) 2D confocal microscopy images of HeLa cells upon exposure to 1000 nM of T22-GFP-H6 or T22-GFP-TD-H6 for 2 h. Red signal indicates labelled membranes, blue signal nuclear nucleic acids and green signal is from the green fluorescent protein. Left panels show green and blue channels of a particular plane, adding the red channel at the right panel. (**B**) 3D reconstructions of confocal stacks along the *z*-axis, at the same conditions. A clipping plane inset is shown for the represented cell exposed to T22-GFP-TD-H6, to indicate the distribution of GFP dots inside the cell. Bars indicate 20 μm.

The dramatic protein accumulation linked to the incorporation of TD (note that T22-GFP-H6 and T22-GFP-TD-H6 differ only in this domain) was assessed by confocal microscopy. The images confirmed the higher levels of T22-GFP-TD-H6 when compared to T22-GFP-H6 (Figure 3A). While an important part of the protein was found intimately associated to the cell membrane, 3D confocal reconstructions revealed an intracellular localization of an important amount of material, differential when comparing protein versions (Figure 3B). Such localization was compatible, in both cases, with an endosomal localization of the material. Since no diffused fluorescence was observed in the cytoplasm, cytosolic release, if it occurred, was moderate and involved only a limited fraction of the engulfed protein. Again, cells did not show any sign of toxicity when exposed to any of these proteins (Figure 3A).

**Figure 4:**
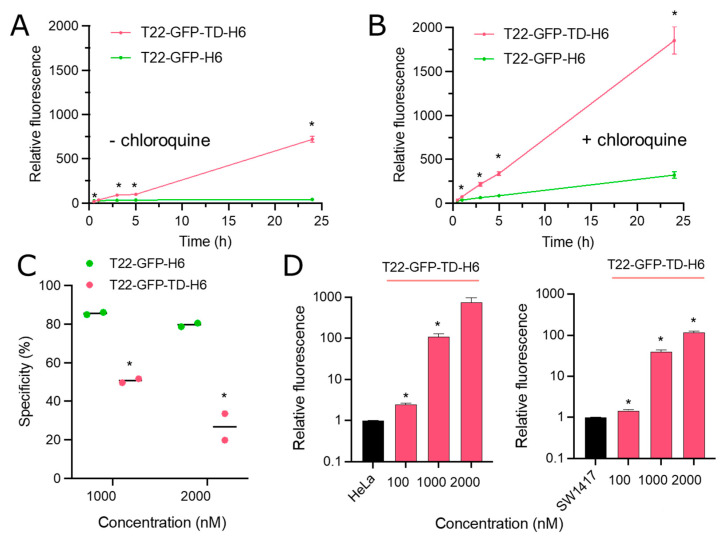
Cell interactivity and fate of T22-GFP-TD-H6 nanoparticles. (**A**) Time-dependent intracellular accumulation of T22-GFP-H6 and T22-GFP-TD-H6 in HeLa cells. Both proteins (stored in 166 mM NaHCO_3_, 333 mM NaCl, pH 8.0) were incubated at 2000 nM. (**B**) Intracellular accumulation kinetics of T22-GFP-H6 and T22-GFP-TD-H6 with chloroquine (0.5 M) incubated 4 h before protein addition. For comparison purposes, conditions tested were the same as in A. For both (**A**,**B**), fluorescence values are referred to the background of non-treated cells. Data are shown as mean ± standard error. Significant statistical differences between data pairs (T22-GFP-H6 vs. T22-GFP-TD-H6 at a respective time) are represented by * at *p* < 0.05. (**C**) CXCR4-specificity of 1000 nM and 2000 nM T22-GFP-H6 or T22-GFP-TD-H6 in HeLa cells, measured by cell internalization after 1 h of exposure. Cells were previously incubated with the CXCR4 antagonist AMD3100 at ten-fold the protein concentration (10 µM or 20 µM). Data are shown as mean ± standard error. Statistical significance between T22-GFP-H6 and T22-GFP-TD-H6 specificity is represented by * *p* < 0.05. (**D**) Intracellular fluorescence of CXCR4^+^ HeLa cells and CXCR4^−^ SW1417 cells exposed to T22-GFP-TD-H6 for 24 h. Fluorescence values are referred to the background of non-treated cells. Data are shown as mean ± standard error. Significant statistical differences between values at each condition and background fluorescence of untreated cells are represented by * at *p* < 0.05.

The potential of TD to enhance the endosomal escape of T22-GFP-H6 was further tested by using chloroquine as an indicator drug. Chloroquine minimizes lysosomal destruction of proteins by reducing acidification [58,59]. Subsequently, any increase in the intracellular florescence of the drug should be representative of the nominal proteolytic destruction of a given fluorescent polypeptide, because of its retention in the lysosome. As observed in kinetic internalization analyses (Figure 4A,B), chloroquine increases the intracellular amount of both T22-GFP-H6 and T22-GFP-TD-H6, indicating lysosomal proteolysis in both cases. Although slight differences between the internalization patterns of these proteins were observed in the drug, data were not sufficiently robust to infer an enhanced proteolytic stability in the TD-carrying protein. Again, according to the data shown in Figure 3, endosomal escape, if it occurred, was not a majoritarian event.

The absence of an evident endosomal disruption (Figure 4A,B) combined with the enhanced intracellular accumulation promoted by TD (Figure 1D and Figure 3A) prompted us to explore the cell-binding specificity of T22-GFP-TD-H6. Furthermore, we wanted to evaluate if TD might alter the exquisite receptor specificity of T22. In this context, contrary to what happened in the case of T22-GFP-H6, the uptake of an important fraction of T22-GFP-TD-H6 was not dependent on CXCR4. This was inferred from the fact that the CXCR4 antagonist AMD3100 blocked the penetration of 80% of T22-GFP-H6 nanoparticles but only 27% of T22-GFP-TD-H6 nanoparticles (data at 2000 nM, Figure 4C). Importantly, data about intracellular fluorescence were obtained after a harsh trypsin cell treatment, which was specifically developed to remove externally attached protein ([54], as described in methods section). In addition, T22-GFP-TD-H6 nanoparticles accumulated in a similar dose-dependent fashion in both CXCR4^+^ HeLa cells and CXCR4^−^ SW1417 cells (Figure 4D). Both set of results support the conclusion that the higher amount of T22-GFP-TD-H6 nanoparticles in target cells compared to parental T22-GFP-H6 nanoparticles was mostly due to a loss of receptor specificity and enhanced cell internalization rather than an apparent and massive endosomal escape.

## 4. Discussion

The DT is one of the most well-known microbial toxins. Through its TD, this protein uses the endosomal pathway of cellular entrance [28]; this domain might be highly appealing as a functional component when designing protein drugs, for which lysosomal degradation is a main bottleneck [15,16,17,18,64]. However, the behaviour of this protein segment as an agent to functionalize protein drugs with refined receptor-mediated targeting [65] has been essentially neglected. In this study, by incorporating the DT TD into T22-GFP-H6 (Figure 1), a protein nanoparticle targeted to CXCR4-overexpressing cells [36], we have demonstrated an enhanced accumulation of the material in comparison to the parental T22-GFP-H6 in dose-dependent (Figure 2D) and in time-dependent (Figure 4A) ways. Considering both types of analyses and the similarity of the specific fluorescence of both constructs (Figure 2E), the amount of intracellular material differed up to around 50-fold (Figure 2D). However, the obtained data were not supportive of an enhanced endosomal escape and consequent proteolytic stability; in contrast, this indicated a nonspecific cell penetrability of nanoparticles empowered with TD, independent of endosome formation. Two observations support this concept. First, the penetration of T22-GFP-TD-H6 is only partially dependent on the receptor CXCR4, to which the peptide T22 binds (Figure 4C). Also, the protein accumulates in a similar pattern in both CXCR4^+^ and CXCR4^−^ cultured cells (Figure 4D), showing only partial selectivity and cell discrimination based on the receptor to which T22 binds [40,66]. In this regard, it is known that recombinant DT TD versions destabilize different types of lipid membranes at neutral or quasi-neutral pH values, namely in the 3–6.5 range [67], and the translocation mechanism of this toxin domain is comparable to that of some cell-penetrating peptides [68]. Therefore, the notable intracellular accumulation of T22-GFP-TD-H6 nanoparticles, in contrast to the parental version lacking TD (Figure 2C), can be accounted for by a receptor-independent, non-specific cell uptake that might involve a large fraction of the presumed cell-targeted material. Like other endosomal escape peptides whose activity is based on the physical modification of the membrane structure [15], the potential of the DT TD in enhancing nanoscale drug delivery might be incompatible with precision medicines that rely on an exquisite receptor recognition.

## 5. Conclusions

The translocation domain (TD) of the DT was incorporated in a GFP-containing protein-only nanoparticle that selectively binds to and internalizes CXCR4^+^ cells through the highly specific ligand T22. In this context, the TD dramatically increased the amount of protein material that accumulate intracellularly. However, this event occurred at the expense of receptor selectivity in the delivery process, which was significantly reduced in the material functionalized with TD. This fact prevents us from considering DT TD as a useful candidate for cell drug delivery in the context of protein-based precision medicines, for which exquisite receptor selectivity is mandatory.

## Data Availability

Raw data can be found here: https://ddd.uab.cat/record/264345, (accessed on 20 November 2022).
